# Insurance Provision for Hospital Workers

**Published:** 1912-01-13

**Authors:** 


### INSURANCE PROVISION FOR HOSPITAL WORKERS.

The National Insurance Act imposes a responsibility upon the managers of all hospitals and kindred institutions to insure those members of the working staff who are affected by the Act. It is only natural that an idea should prevail that the way to secure the best possible result for the insured will be for hospital managers collectively to consider and determine how to secure for their employees admission to an approved society which will offer them the maximum security.

The idea that steps should be taken by the hospitals collectively to form an approved society for the benefit of those institutional workers who come under the Act has been under consideration during the last few weeks. It is not, however, free from difficulties, one of which is that the first plan of forming one organisation to include both sexes is negatived by the Act itself, which provides that the two sexes are to be kept apart as though they belonged to two different societies. The actuaries, we gather, think it possible, however, to have one society with two branches, providing a way can be found to keep the surplus arising from the male and female branches respectively absolutely separate and distinct. In any case, it should be practicable to overcome any difficulty by having two separate societies, one for men and one for women, providing a sufficient number of members can be recruited from the male staff of hospitals to secure a safe average. The Royal National Pension Fund for Nurses and Hospital Officials has kept in the closest touch with Mr. Lloyd George and the Government, so that it has been enabled to prepare the ground for the immediate formation of a separate section of their organisation as an approved society. Everything is, therefore, in a forward condition to enable a working arrangement to be made between every hospital committee and the Royal National Pension Fund, whereby all the employees of a hospital may become insured under the National Insurance Act. The Royal National Pension Fund has invested funds which already exceed one and a half million pounds sterling.

The great point to be determined is, whether it will be best to create one society with two branches or to have two separate societies—*i.e.*, one for each sex. It is essential that everybody should realise that the position of men and women in connection with this insurance question differs materially. Thus the greater portion of the nursing staff of each hospital consists of young women who probably have not yet been insured anywhere, with the possible exception of the Employers' Liability Act, though most of those so insured are probably unaware of the fact. Each hospital may therefore find it a simple matter to deal with the nursing staff by placing it in the hands of the matron or the secretary, or of both in co-operation. On the other hand, the men employed in a hospital may already be connected with some existing insurance company or institution. The need of the moment is for accurate returns to be supplied showing the number of men actually employed in the hospitals throughout Great Britain. We should be much obliged to any secretary who will kindly send us a postcard containing this information, so far as it relates to his institution. It will so be possible to ascertain whether the male employees throughout the hospital world are collectively sufficient in number to form a safe average to work upon. We hope that hospital committees and the Council of the Fund will approach each other and form a committee to thrash out the details of a working basis, and that hospital employees and trained nurses will abstain meanwhile from pledging themselves to support any approved society.

